# Compact TSA with Anti-Spiral Shape and Lumped Resistors for UWB Applications

**DOI:** 10.3390/mi12091029

**Published:** 2021-08-27

**Authors:** Xue-Ping Li, Gang Xu, Chang-Jiao Duan, Ming-Rong Ma, Shui-E Shi, Wei Li

**Affiliations:** 1 College of Electronic and Electrical Engineering, Henan Normal University, Xinxiang 453600, China; gxu2020@126.com (G.X.); cjduan2020@126.com (C.-J.D.); mrma2020@126.com (M.-R.M.); shishuie@htu.edu.cn (S.-E.S.); 2 Henan Key Laboratory of Optoelectronic Sensing Integrated Application, Henan Normal University, Xinxiang 453600, China; 3 Academician Workstation of Electromagnetic Wave Engineering of Henan Province, Henan Normal University, Xinxiang 453600, China

**Keywords:** tapered-slot-fed antenna (TSA), anti-spiral shape, resistor-loading, ultra-wideband (UWB)

## Abstract

A novel compact tapered-slot-fed antenna (TSA) with anti-spiral shape and lumped resistors is presented for ultra-wideband (UWB) applications. Unique coplanar waveguide (CPW) to coplanar strip (CPS) feeding structure and exponential slot are designed to ensure the continuous current propagation and good impedance matching. With a pair of anti-spiral-shaped structure loadings at the end of the antenna, the radiation performance in lower operating band can be enhanced obviously. The typical resistor loading technique is applied to improve the time domain characteristics and expand the bandwidth. The fabricated prototype of this proposed antenna with a size of 53 × 63.5 mm^2^ was measured to confirm simulated results. The proposed antenna has S11 less than −10 dB in the range of 1.2–9.8 GHz, and the group delay result is only 0.4 ns. These findings indicate the proposed antenna can be taken as a promising candidate in UWB communication field.

## 1. Introduction

Ultra-wideband (UWB) technology can be taken as a powerful complement to narrowband technology due to its special characteristics, such as wide bandwidth, low profile, simple manufacturing, moderate gain in compact size, and endfire radiation pattern [1]. It is not only utilized in wireless communication, radar imaging and sounding, but also for human care monitoring and astronomy [2,3,4,5]. For the high integration requirement in the communication field, the volume of a mobile device is changed to lighter and tinier, and a miniature UWB antenna has become an indispensably technique during the communication design. Stripline-fed antenna constructed in the ground plane was the first realization of the tapered slot antenna (TSA). In addition, the first printed slot antenna and a double exponentially tapered slot antenna (DETSA) have been introduced and some conformal studies are also implemented for more promising applications [6,7]. In recent years, lots of different types of tapered slot antennas (TSAs) have been proposed and compared according to its specific application and technology requirement [8,9,10]. For wireless communication, it is important to know how to obtain large bandwidth and high gain through wide impedance matching structure and novel antenna shape in a compact size [11,12]. However, in time domain application, ensuring good signal fidelity is much more important, and some non-reflection optimization and loss loading are deployed to remove the reflected power from the discontinuous points and end truncation [13].

The TSA on a dielectric substrate attracts much attention for its small size, low cost and weight, easy manufacture, and wideband properties. The TSA mainly consists of two different parts, such as tapered radiation slot in coplanar or antipodal geometry and wideband feeding network for unbalance to balance transition between the RF front and the radiated section [14,15]. To achieve good performance for the antenna design, each part should be optimized carefully. In past decades, research on TSA has been published under different backgrounds and applications [16,17,18,19]. Coplanar TSA usually possesses wide bandwidth over two octaves with lower cross-polarization. However, feeding transition from the unbalanced port to the balanced slot faces great challenges [20]. At this moment, microstrip-to-slotline transition is highly used [21]. In contrast to one coplanar TSA, antipodal design can achieve much wider bandwidth due to natural UWB feeding transition, where the vertical E-field in microstrip line can be guided into inclined E-field from the top edge to bottom side of antipodal slot. Generally, the bandwidth of TSA is partly proportional to their length and aperture. In order to overcome this limitation, some modifications about the tapered slot and end truncation tips were implemented. For example, exponential or elliptical tapered slot were analyzed for better continuity and appropriate aperture, and the coplanar waveguide (CPW) to slot transitions were applied for better natural transformation [22,23]. Among these proposed UWB TSAs, the optimal antenna design should be one tradeoff choice according to the required small structure, easy manufacturing and convenient to be integrated with miniature RF front ends.

In this paper, we propose a modified coplanar TSA, which is terminated with two novel anti-spiral shape to enlarge the electrical aperture and reduce the reflection power. As the antenna aperture increases, the width of anti-spiral arms quickly sharpen as two curved horn of the buffalo. To further eliminate the reflections from the end tip, two lumped resistors are inserted at the half of the road path to largely absorb the residual current. Finally, one antenna prototype is fabricated and measured fully, and some analyses are implemented for better understanding the proposed antenna.

## 2. Slot Antenna Configuration and Design Consideration

### 2.1. Antenna Configuration

**Figure 1:**
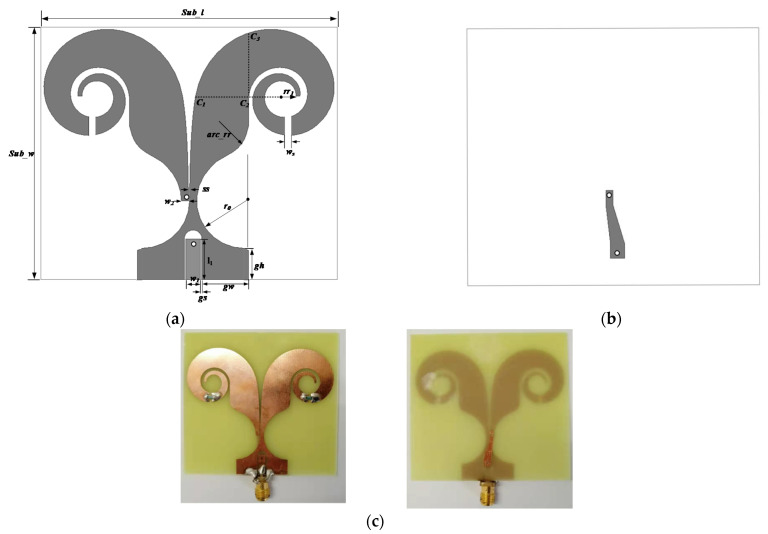
Geometry of the antenna: (**a**) top view, (**b**) bottom view, and (**c**) fabricated antenna model.

In the beginning, an antenna based on the CPW to coplanar strip (CPS) feed is proposed after reviewing some references, and better UWB characteristics and compact dimensions can be achieved by using the exponential equation with spiral curve at the termination of the antenna, in this process, the antenna has the disadvantage of tail current discontinuity and excessive current reflection at low frequencies. Thus, to obtain good fidelity of radiation signal and expand the low frequency characteristics of the antenna bandwidth, a resistor loading operation is performed at the end of the antenna. Thus, the final structure of the antenna is obtained. Figure 1a,b demonstrates the geometry of the proposed antenna, and the actual antenna prototype is shown in Figure 1c. It is notable that Figure 1a,b is just an illustrative image with the symbolic mark and position. The original antenna is constructed on an FR4 substrate. To obtain wider bandwidth and good signal fidelity, two lumped resistors in PCB footprint of 1210 are chosen. Meanwhile, to ensure the path continuity and smoothness, some radial fillet operation is made at the corner. The optimal dimensions of modified coplanar TSA are listed in Table 1.

The proposed antenna includes three independent parts, the CPW to CPS transition, the exponentially tapered slot, and the loaded anti-spiral shape. One polyline strip is introduced to transfer the unbalanced field in CPW into the balanced field in CPS. Below the dashed line C_1_ to C_2_, one exponential slot is designed to ensure the continuous current propagation and good impedance matching. The according exponential equation can be represented as Equation (1). The final anti-spiral loaded with the exponential part for frequency-independent characteristics at low frequency band can be described by Equation (2). In addition, to make good signal fidelity, two lumped resistors are inserted at the half path of the spiral.

(1) $$y(x)=ae^{kkx}+b\;0\;\mathrm{mm}\leq x\leq 40\mathrm{mm}$$

(2) $$\left\{ \begin{array}{l} x(\theta )=rr_{1}e^{k_{0}\theta }\cos(\theta )\\ y(\theta )=rr_{1}e^{k_{0}\theta }\sin(\theta )\;0\leq \theta \leq 3\pi \end{array}\right.$$

where *kk* demonstrates the degree of the exponential curves. *a* and *b* are related to the length and width of the arms. $rr_{1}$ is the length of starting inner radius of the spiral curve and *θ* is the rotation angle of the spiral curve.

### 2.2. Parameter Study

**Figure 2:**
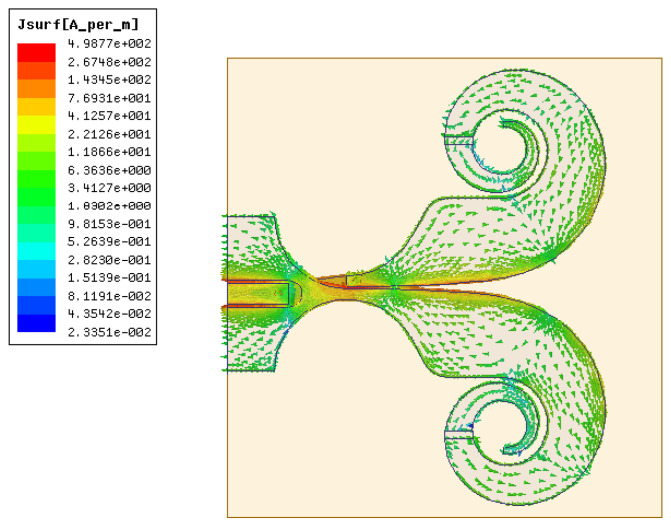
Simulated current distribution on the proposed antenna surface at 5.5 GHz.

To investigate the influence of the critical parameters on the electrical characteristics of the proposed antenna, a parametric study has been carried out. The simulated current distribution for the designed antenna on the center frequency point of 5.5 GHz is depicted in Figure 2. As the power propagation and radiation along the main slot, the residual current become small and low at the end, which illustrates the good impedance bandwidth and fidelity.

**Figure 3:**
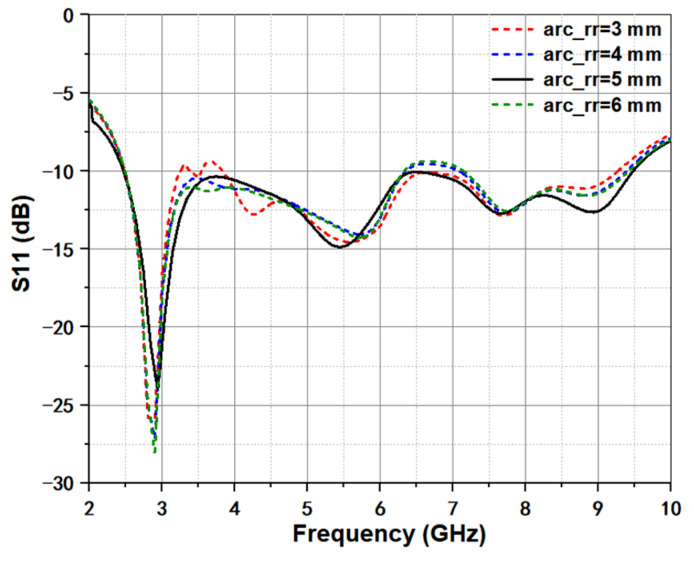
Simulated return loss under different fillet radius arc_rr.

To evaluate the effect of fillet radius arc_rr, the size is increased from 3 to 6 mm with an interval of 1 mm. The response is shown in Figure 3. It can be observed that the arc_rr had little effect on the return loss. In fact, the main function for the fillet operation is to smooth the current path and eliminate some strong reflection points. Therefore, all of these curves have nearly identical shape and value among the whole frequency band.

**Figure 4:**
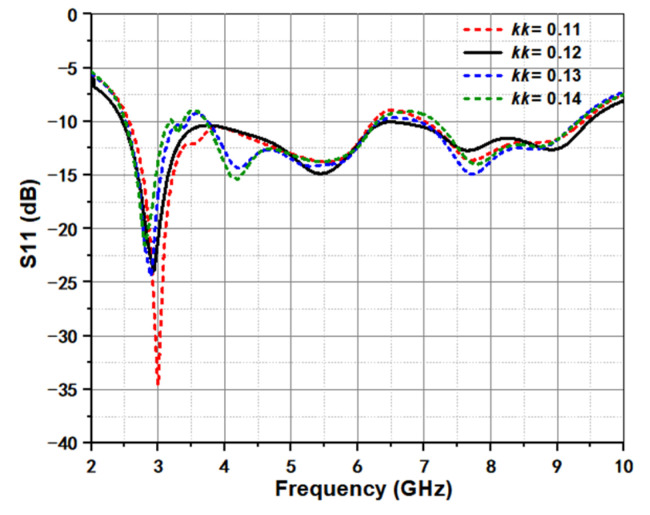
Simulated return loss under different exponential index *kk*.

**Figure 5:**
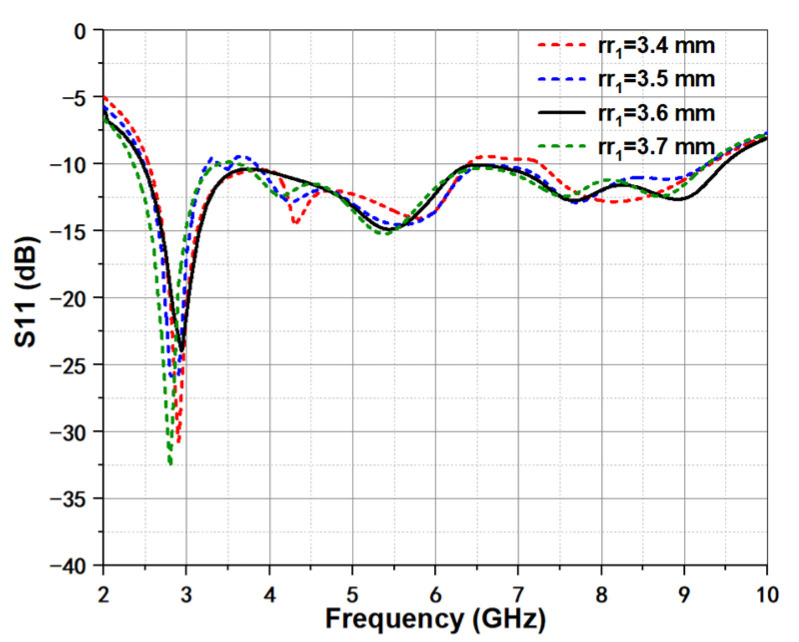
Simulated return loss under different initial radius $rr_{1}$ of spiral curve.

Figure 4 shows the effect of exponential index *kk* on return loss. It is observed that the exponential index *kk* can affect the return loss, which is because that different tapered slot can induce the energy transition quality from feeding structure to radiated aperture unequal. As the value of *kk* increases, the return loss at 3.5 GHz also gradually increases, while the return loss at 6.5 GHz exhibits a decreasing trend. The same effect of initial radius $rr_{1}$ in spiral can be seen in Figure 5. For spiral curve, small $rr_{1}$ can quickly produce large difference at the exponential speed. Though the value only changes from 3.4 to 3.7 mm with a small step of 0.1 mm, the return loss between them exhibits different appearances. Moreover, $rr_{1}$ is the design parameter of the spiral radiator in the termination of antenna, the main difference brought by the change of this parameter is located at the joint position C_1_ to C_2_. Thus, for good bandwidth, to ensure the continuous current propagation path is important in design process.

**Figure 6:**
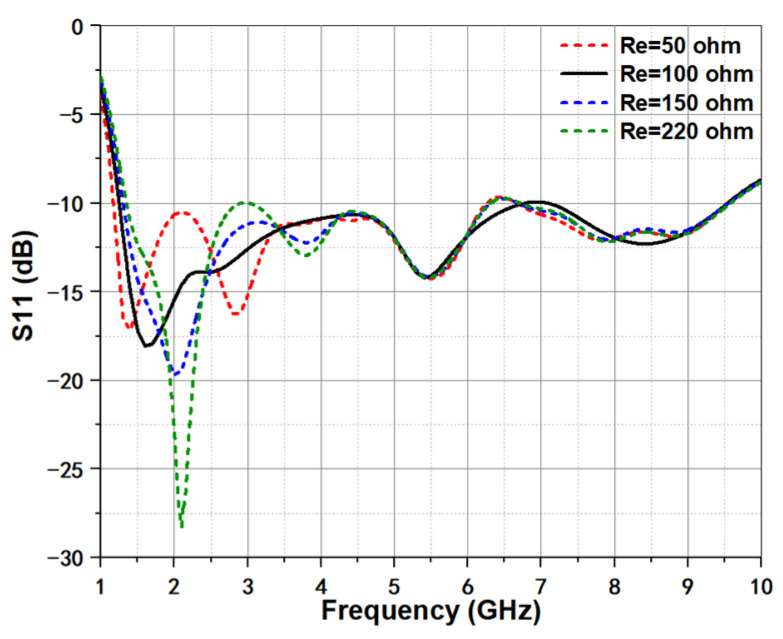
Simulated return loss under different loaded resistors.

For resistive loading, the primary role is to absorb the residual current at the low frequency band, so some evident changes should be seen at lower band. In Figure 6, we can find that the loaded resistor has little effect on the higher band, and nearly identical curves are kept. However, significant difference can be observed at lower band. Meanwhile, under different values, the lower cutoff frequency point is same but the variation tendency is widely different. When the loaded resistor value is 100 Ω, the return loss has better resonance status.

**Figure 7:**
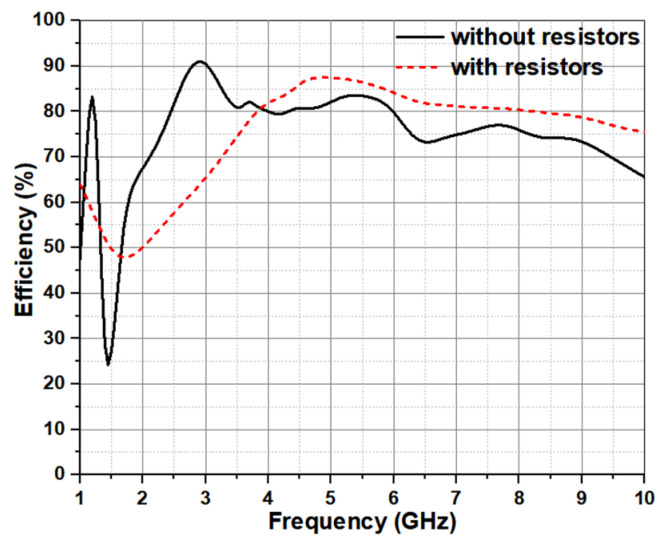
Effect of the resistors on radiation efficiency.

As is known, the resistive loading in the antenna can influence the radiation characteristics (see Figure 7). The simulated antenna efficiency before loading the resistor is about 70% to 90%, but there is a trap frequency around 1.5 GHz. By adding the resistive loading in the antenna, the radiation efficiency at low frequencies has been improved obviously, and at high frequencies can be enhanced except the range of 1.65–3.85 GHz. In addition, the maximum difference of insertion loss between them can be up to 1 dBi at 3 GHz.

## 3. Results and Discussion

**Figure 8:**
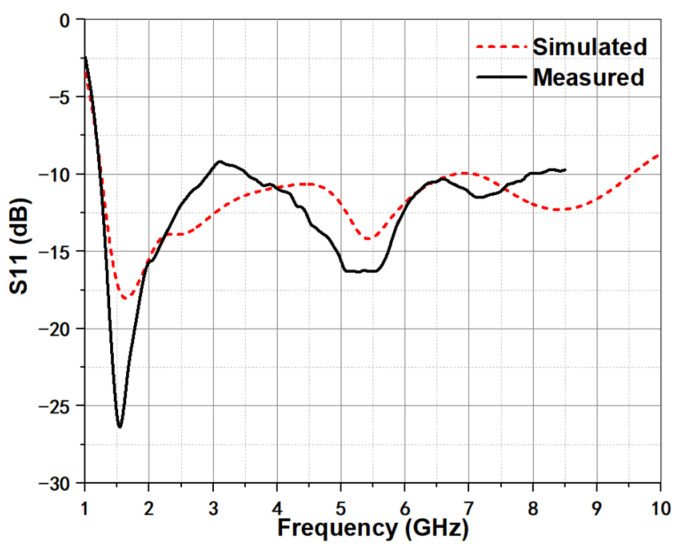
Simulated and measured return loss of compact UWB TSA.

The compact UWB TSA is projected and optimized by two different 3-D full-wave electromagnetic simulation tools of Ansoft HFSS and CST, and the relevant parameters, such as return loss, radiation patterns, and group delay are measured by the Agilent vector network analyzer (VNA) E5071C (Keysight Technologies, Inc., Santa Rosa, DE, United States). Figure 8 displays the measured and simulated return loss of the proposed antenna, and the measurement is implemented just up to 8.5 GHz subject to VNA E5071C. The simulated impedance bandwidth with return loss better than −10 dB is 1.2–9.8 GHz. Moreover, the simulation and measurement agree well with each other. The small discrepancy between them is induced by the introduction of SMA connector and construction tolerance.

**Figure 9:**
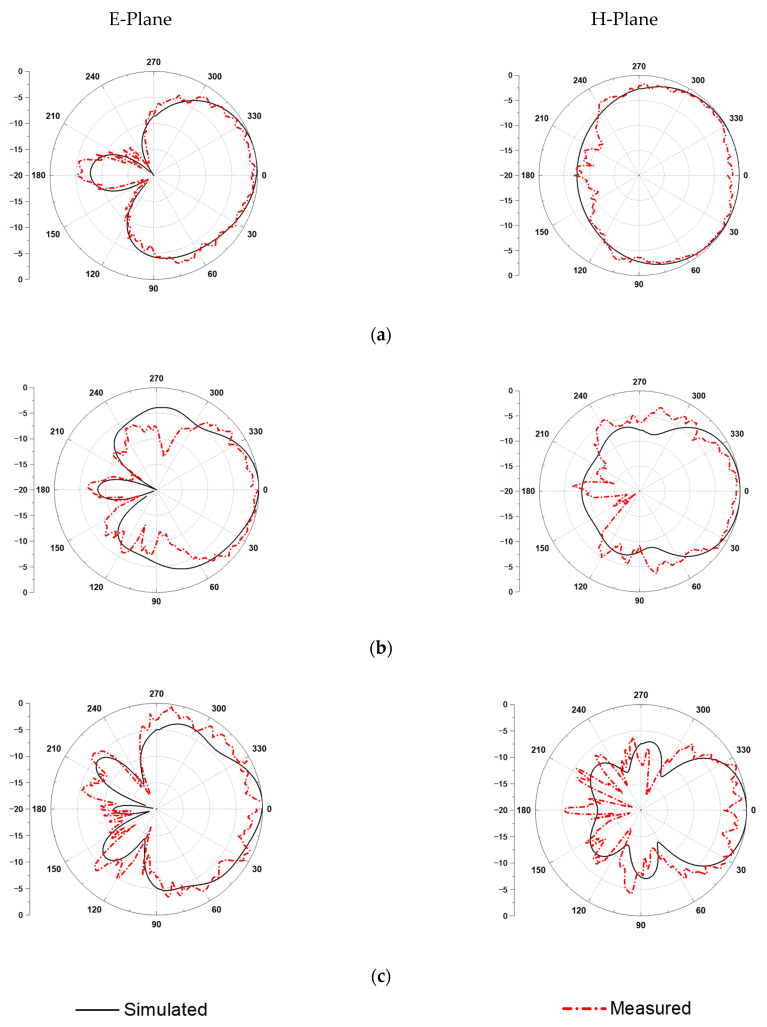
Simulated and measured radiation patterns of E-plane and H-plane: (**a**) 3 GHz, (**b**) 5 GHz, and (**c**) 7 GHz.

In addition, the normalized simulated and measured radiation patterns of 3, 5, and 7 GHz are exhibited in Figure 9. The radiation of the E-plane and H-plane shows good endfire characteristics in the working frequency band, and the simulation is well consistent with the measured results.

**Figure 10:**
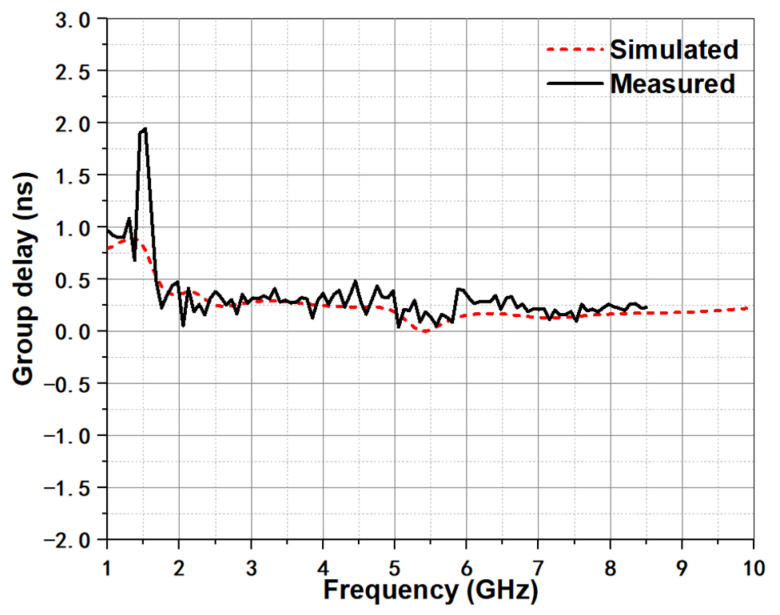
Simulated and measured group delay of compact UWB TSA.

For good signal fidelity, group delay is one important index in time domain. Analyzed from the linear system perspective of the signal system, the delay fluctuation at each frequency point of group delay is equivalent to the phase change of the signal in this system, while the average constant of group delay is equal to time delay of the signal, which means the response to the signal is ahead or lagging. Thus, two antennas face-to-face 500 mm apart are built and then obtained the simulation results of group delay characteristics. In the actual measurement, the VNA E5071C with time domain analysis function was used for the tests. Figure 10 demonstrates the full comparison between measured and simulated group delay. It can be seen that the group delay of proposed antenna is around 0.4 ns. There exist agreement in measurement and simulation. In the working band, only 0.4 ns fluctuation can be seen, which largely ensured the in-band linear smoothness.

**Figure 11:**
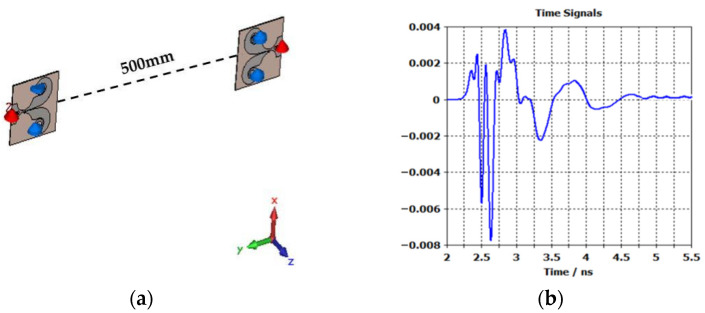
Conformal antenna and its performance in time domain: (**a**) conformal antenna and (**b**) received signal.

For many UWB applications, the antenna performance in time domain is very critical, which can largely influence the radiated signal quality in transceiver. To understand the ability for the proposed antenna, a pair of antennas is constructed face-to-face with the distance of 500 mm at identical height by CST, see Figure 11a, and one narrow Gaussian pulse is set as excited source with operating bandwidth from DC to 10 GHz. The final received signal is shown in Figure 11b. Large bandwidth and good input characteristics ensure very small ringing tails at the end of main wave.

Furthermore, Table 2 lists the comparison of presented antenna with other existing studies. Though the antenna proposed in [24,25,26,27] is comparatively smaller than that of this work, it is observed that the fractional bandwidth of the proposed antenna is relatively larger than the fractional bandwidth reported by the authors and the gain is comparable. The size and fractional bandwidth of the proposed antenna in this work is superior to that of the reported work in [28,29,30], as shown in Table 2. Although, the proposed antenna in [31,32,33,34] possess better gain and fractional bandwidth; nonetheless, with larger size, more expensive substrate, and worse group delay property. It can be observed that the proposed antenna has the advantages in compact size, fractional ultra-wide bandwidth, low cost, and good enough merit of gain. Therefore, it can be well suited for high-speed UWB applications.

## 4. Conclusions

A Compact TSA with a dimension of 53 × 63.5 mm^2^ for UWB applications is proposed and implemented in this paper. In the designed antenna, a unique CPW-CPS feeding structure and exponential slot are utilized. In addition, the final anti-spiral shape is loaded with the exponential part for frequency-independent characteristics and two lumped resistors are inserted at the half path of the spiral. A measured operating band from 1.2 to 9.8 GHz for S11 < −10 dB is obtained. The group delay result is only 0.4 ns and its up and down fluctuation is not more than 0.4 ns. The measurement is proved to be in accordance with simulation, which makes this antenna suitable for UWB communication systems.

## Figures and Tables

**Table 1:** Optimal dimensions (mm) and loaded resistors (Ω) of modified coplanar tapered slot antenna (TSA).

Parameter	sub_l	sub_w	ss	gs	gh	gw	l_1_	w_1_	w_2_	w_s_	rr_1_	r_0_	arc_rr	Resistors
Unit	62	53	0.2	0.35	6.6	9.78	8.6	3	1.5	1	3.6	10	5	100

**Table 2:** Comparison of the proposed and reference antennas.

Ref.	Physical Size (mm^2^)	Electrical size (λ_0_^2^)	Substrate	Bandwidth (GHz)	Gain (dBi)
[24]	38.3 × 34.5	0.33 × 0.3	FR4	121% (2.6–10.58)	2.5–5
[25]	40 × 45	0.31 × 0.35	Rogers Duroid TM	144.7% (2.34–14.6)	2.25–7.75
[26]	64 × 37.4	0.32 × 0.19	F4BM	149.6% (1.5–10.4)	2–7
[27]	48 × 60	0.5 × 0.6	FR4	141.5% (2.4–14)	3.7–10
[28]	98 × 110	0.46 × 0.51	FR4	148% (1.45–9.8)	6.28
[29]	100 × 78	0.67 × 0.52	Taconic TLY-5	86% (2–5)	3–5.19
[30]	66.4 × 50	0.89 × 0.67	$\varepsilon_{r}=4.5$	153% (4–30)	5–7.5
[31]	450 × 600	0.45 × 0.6	Rogers 4350	147.8% (0.3–2)	4–11.5
[32]	90 × 93.5	0.4 × 0.42	F4B	171% (1.32–17)	3.5–9.3
[33]	158 × 125	0.38 × 0.3	Taconic TLT	183.7% (0.72–17)	1–12.5
[34]	161 × 140	0.45 × 0.39	$\varepsilon_{r}=2.3$	174% (0.83–9.8)	2.5–10.4
This work	53 × 63.5	0.21 × 0.25	FR4	156.4% (1.2–9.8)	4–5.2
